# Severity classification of repeated isoflurane anesthesia in C57BL/6JRj mice—Assessing the degree of distress

**DOI:** 10.1371/journal.pone.0179588

**Published:** 2017-06-15

**Authors:** Katharina Hohlbaum, Bettina Bert, Silke Dietze, Rupert Palme, Heidrun Fink, Christa Thöne-Reineke

**Affiliations:** 1 Institute of Animal Welfare, Animal Behavior and Laboratory Animal Science, Department of Veterinary Medicine, Freie Universität Berlin, Berlin, Germany; 2 Institute of Pharmacology and Toxicology, Department of Veterinary Medicine, Freie Universität Berlin, Berlin, Germany; 3 Unit of Physiology, Pathophysiology and Experimental Endocrinology, Department of Biomedical Sciences, University of Veterinary Medicine, Vienna, Austria; Massachusetts General Hospital, UNITED STATES

## Abstract

According to the EU Directive 2010/63, the severity of a procedure has to be classified as mild, moderate or severe. General anesthesia is thought to be mild, but the Directive does not differentiate between single and repeated anesthesia. Therefore, we investigated the impact of repeated administration of isoflurane, the most commonly used inhalation anesthetic, on the well-being of adult C57BL/6JRj mice, in comparison to single administrations and to untreated animals, when applied six times for 45 min at an interval of 3–4 days. For the animals anesthetized, excitations, phases of anesthesia, and vital parameters were monitored. Well-being after anesthesia was assessed using a behavioral test battery including luxury behavior like burrowing and nest building behavior, the Mouse Grimace Scale (MGS), the free exploratory paradigm for anxiety-related behavior, home cage activity and the rotarod test for activity, as well as food intake and body weight. Additionally, hair corticosterone and fecal corticosterone metabolites were measured. Our results show that nest building behavior, home cage activity, body weight, and corticosterone concentrations were not influenced by anesthesia, whereas changes in burrowing behavior, the MGS, food intake, and the free exploratory behavior indicated that the well-being of the mice was more affected by repeated than single isoflurane anesthesia. This effect depended on the sex of the animals, with female mice being more susceptible than male mice. However, repeated isoflurane anesthesia caused only short-term mild distress and impairment of well-being, mainly in the immediate postanesthetic period. Well-being stabilized at 8 days after the last anesthesia, at the latest. Therefore, we conclude that when using our anesthesia protocol, the severity of both single and repeated isoflurane anesthesia in C57BL/6JRj mice can be classified as mild. However, within the mild severity category, repeated isoflurane anesthesia ranks higher than single isoflurane anesthesia. Additionally, our results imply that male and female mice can differently perceive the severity of a procedure.

## Introduction

In laboratory animal science, the most commonly used inhalation anesthetic in rodents is isoflurane [1]. Its use has increased over the last years [2] because of its quick on- and offset of anesthesia and low metabolism rate [3]. Isoflurane causes moderate respiratory and cardiovascular system depression, but maintains better cardiac function than the combination of ketamine and xylazine [3]. However, apart from these advantages, isoflurane was also found to cause reversible deficits in object recognition memory [4], impaired learning function in the cued fear conditioning, and higher anxiety-related behavior in the elevated plus maze in mice in the postanesthetic period [5]. Rabbits even have periods of apnea during the induction of anesthesia with isoflurane indicating a high aversion towards this inhalant agent [6]. In mice and rats, repeated administration of isoflurane is more aversive than a single administration [7]. Additionally, general anesthesia in rodents is often associated with several side effects, e.g. the disturbance of the circadian rhythm [8] as well as hypothermia and hypoglycemia [9], which can negatively influence the recovery period.

Isoflurane anesthesia is repeatedly performed in studies using imaging techniques for small animal models [10]. In order to apply imaging techniques correctly and to gain the optimum results, animals need to be immobilized by using general anesthesia [11]. Imaging techniques are non-invasive and contribute to reducing the number of animals for an experiment, since several experiments are conducted using a single animal. Instead of euthanizing an animal at each time point of the study, an animal can be examined several times in its life in order to control the progress of diseases like stroke, tumor growth, bone healing, epileptogenesis or neurodegenerative disorders. Therefore, imaging techniques are a great benefit for reduction and refinement. However, in terms of the 3R-principle the benefit of the repeated use of an animal only becomes obvious, when the total amount of pain, distress or harm does not exceed the severity degree of pain, distress or harm of each single manipulation. Hence, with regard to the EU Directive 2010/63 [12], this needs to be evaluated for every test strategy that shall be applied in an animal. The Directive implies to fully apply the 3-R-principle of Russel and Burch [13], i.e. not only to replace animal experiments, but also to reduce the number of animals and to refine animal experiments, whenever they are necessary. Moreover, the severity of a procedure has to be classified as mild, moderate or severe. According to Annex VIII of the EU Directive 2010/63, general anesthesia is considered as mild, though, the Directive does not distinguish between a single and repeated anesthesia. However, in fact, it is unknown whether repeated anesthesia affects well-being in the same way as single anesthesia. We hypothesized that repeated anesthesia may enhance the behavioral and pharmacological effects of isoflurane in laboratory animals and, subsequently, causes additional distress for the animal.

In order to test our hypothesis, we investigated the effects of repeated compared to single isoflurane anesthesia on the well-being of mice. Based on this, we assessed the severity category of repeated isoflurane anesthesia. We chose C57BL/6JRj mice, since they are widely used in research studies [14], and examined both sexes.

In the present study, mice were anesthetized six times for 45 min at an interval of 3–4 days over 3 weeks according to Albrecht et al. [15, 16], which is equivalent to anesthesia protocols performed in imaging studies. Although surgical tolerance is not a mandatory prerequisite for imaging techniques, we induced surgical tolerance, so that the results can also be transferred to studies including repeated minor invasive manipulations like blood or tissue sampling [17]. Well-being was assessed by a broad behavioral test battery in the postanesthetic period and non-invasive analysis of the adrenocortical activity.

## Methods

### Ethics statement

The study at hand was performed according to the guidelines of the German Animal Welfare Act and was approved by the Berlin State Authority (“Landesamt für Gesundheit und Soziales”, permit number: G0053/15). A sample size calculation was performed to determine the number of animals to be used. Experimental methods were refined by closely monitoring the animals after anesthesia. The duration of single housing to measure specific parameters, i.e. nest building behavior, home cage activity, food intake, and fecal corticosterone metabolites (FCM), was kept to a minimum.

### Animals and handling methods

A total number of 32 adult female and 33 adult male C57BL/6JRj mice at the age of 11–13 weeks obtained from Janvier Labs (Saint-Berthevin Cedex, France) were used. The mice were randomly assigned to the 6 study groups: control ♀ (n = 6), control ♂ (n = 7), single anesthesia ♀ (n = 13), single anesthesia ♂ (n = 13), repeated anesthesia ♀ (n = 13), repeated anesthesia ♂ (n = 13). Female mice were group-housed with 3–5 mice in Makrolon type IV cages (55 × 33 × 20 cm). Male mice had to be single-housed in Makrolon type III cages (42 × 26 × 15 cm) with the beginning of the experiments due to aggressive behavior against conspecifics. The cages contained fine wooden bedding material (LIGNOCEL® 3–4 S, J. Rettenmaier & Söhne GmbH + Co. KG, Rosenberg, Germany). A red plastic house, tunnels, and nestlets (Ancare, UK agents, Lillico) were provided as cage enrichment. The animals were maintained under standard conditions (room temperature 22 ± 2°C; relative humidity 55 ± 10%) on a light:dark cycle of 12:12 hours of artificial light with a 5 min twilight transition phase (lights on from 6:00 a.m. to 6:00 p.m.). The mice were fed pelleted mouse diet (Ssniff rat/mouse maintenance, Spezialdiäten GmbH, Soest, Germany) and had free access to tap water.

In order to prevent the impact of distress caused by male persons, both animal care attendant and veterinarian were female [18]. The same veterinarian performed all experiments. A week before the experiments started, the mice were habituated to handling by using combined tunnel and cup handling. The mice were carefully caught in a tunnel belonging to the standard enrichment and then transferred to the experimenter’s hands. This method is known to cause less anxiety in mice than picking up the mice by the tail [19].

### Test schedule

**Fig 1:**
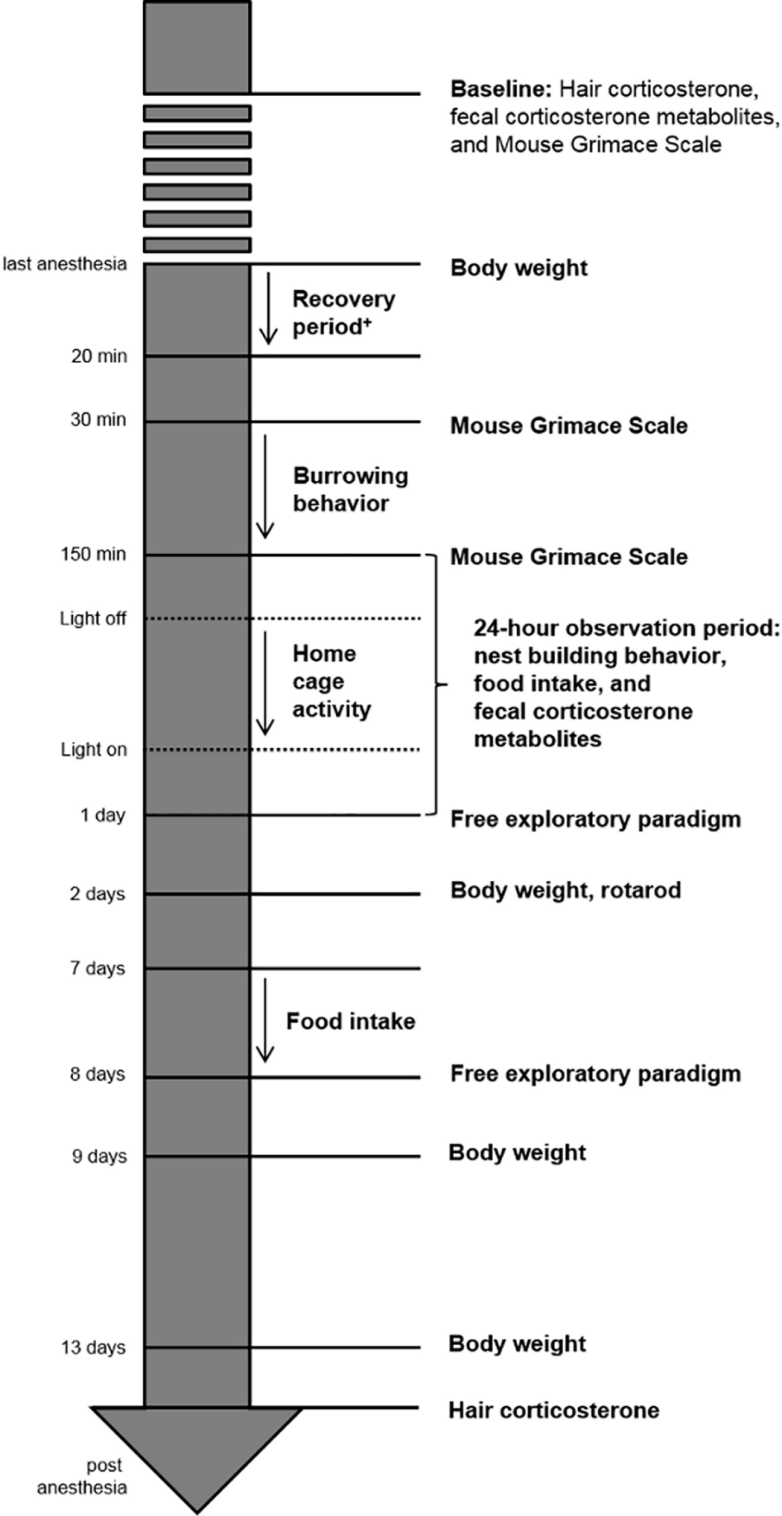
Flow chart of the test schedule. ^+^ Number [n] of rearings, number [n] of grooming episodes, duration of resting [s], duration of activity [s], duration of food intake [s], and the latency to first food intake [s] were observed during the recovery period.

The test schedule is outlined in Fig 1. At the beginning of the experiment, photos for the baseline Mouse Grimace Scale (MGS) score were taken and samples for baseline values of FCM as well as hair corticosterone were collected.

Anesthesia was performed as follows: mice of the group with single anesthesia were anesthetized once for 45 min, mice of the group with repeated anesthesia were anesthetized 6 times for 45 min at an interval of 3–4 days over a period of 3 weeks, and control mice did not receive any anesthesia.

During the induction and after the cut-off of narcosis, the loss and regain of reflexes were observed in order to determine the phases of anesthesia. During anesthesia, vital parameters (i.e. heart rate, oxygen saturation, and respiratory rate) were carefully monitored. After anesthesia, the mice were directly transferred to a custom-made box and their behavior was monitored for 20 min. Photographs of the mouse faces for applying the MGS were taken 30 min after the last anesthesia. 150 min after awakening another photo was taken. In the meantime, burrowing behavior was tested for 2 h. Then all mice were transferred into a (new) Makrolon type III cage and single-housed for a period of 24 hours in order to evaluate nest building behavior, to measure food intake, and to collect fecal samples for FCM analysis. Moreover, home cage activity was recorded for 12 h during the dark period. In order to observe a change in anxiety-related and exploratory behavior over time, the free exploratory paradigm was performed at 1 day and 8 days after the last anesthesia. The rotarod test for motor coordination and balance was conducted at 2 days after last anesthesia. Hair samples for corticosterone measurement were collected at the beginning and at the end of the study, when hair had regrown. Mice were regularly weighted over the whole study period.

### Anesthesia

Anesthesia was induced with 4% isoflurane (Isofluran CP®, CP-Pharma Handelsgesellschaft mbH, Burgdorf, Germany) in 100% oxygen in an anesthetic chamber (with sliding cover, Evonik Plexiglas, 240 × 140 × 120 mm), which was not prefilled in order to prevent distress. During induction, the number of mice showing excitations was recorded.

Anesthesia was divided in five anesthetic stages [15]: 1) Induction started when isoflurane was turned on and ended with the loss of righting reflex. 2) Non-surgical-tolerance was defined as the time from loss of righting reflex to loss of pedal withdrawal and lid reflex. 3) Surgical tolerance began with the loss of all reflexes tested and ended when isoflurane administration was terminated after 45 min. 4) Then the wake-up period followed and ended after the first forward movement. 5) Recovery period was defined as the time from the first forward movement until 20 min after anesthesia.

In order to test the loss of righting reflex, the anesthetic chamber was tipped over. After loosing their righting reflex, the mice were laid in abdominal position on a heating pad and anesthesia was maintained with 1.75–2.5% isoflurane in 100% oxygen via nose cone for 45 min. Artificial tears (Artelac® Splash MDO®, Bausch & Lomb GmbH, Berlin, Germany) were administered to both eyes to prevent the eyes from drying out. When pedal withdrawal and lid reflex were lost, all reflexes were regularly tested. 45 min after induction, isoflurane administration was terminated and the latency to forwards movement, the number of mice showing excitations and/or opisthotonus during wake-up period and mice showing twitches (skin fasciculation) during recovery period were observed.

During anesthesia, vital parameters (respiratory rate, heart rate, and oxygen saturation) were controlled every 10 min. Respiratory rate was counted for 15 sec and then calculated for 1 min [breathes per min]. A pulse oximeter (MouseOx, STARR Life Sciences® Corp., Oakmont, PA, USA), attached to the shaved left hind leg, was used to measure heart rate [beats per min] and oxygen saturation [%].

### Recovery period

The mice were video-recorded for 20 min after the first forward movement in the custom-made photography cube. Number [n] of rearings, number [n] of grooming episodes, duration of resting [s], duration of activity [s], and duration of food intake [s] were analyzed with ethological analyses software (Etholog version 2.2.5; Ottoni 1999). Furthermore, the latency to the first food intake [s] was noted. Activity comprised forward movement and sniffing behavior. Grooming behavior was defined as self-grooming including paw licking, nose and face wash, head wash, body wash and fur licking, leg licking, and tail/genitals licking and wash [20].

### Mouse Grimace Scale (MGS)

The MGS was originally developed to assess pain [21]. However, besides pain, stress has also an impact on the MGS [21] and, moreover, positive emotions are indicated by facial expressions [22]. Thus, in the present study, the MGS served as a tool to assess distress. Scores were obtained by photographs taken in a custom-made box used as photography cube (with three white and one clear wall, 22 × 29 × 39 cm, 0.5 cm bedding material) at 2 days before the first anesthesia (baseline), 30 min after the last anesthesia and 150 min after the last anesthesia. For photography, a high definition camera (Canon EOS 350D, Canon Inc., Tokyo, Japan) was used.

The photograph was cropped to display only the head of the mouse so that the body position was not visible [21]. According to Langford et al. [21], five facial action units, i.e. orbital tightening, nose bulge, cheek bulge, ear position, and whisker change, were scored on a scale from 0 to 2 (0 = not present, 1 = moderately present, 2 = obviously present).

Three blinded persons independently analyzed the photographs. For each scorer, the mean of the five facial action units was calculated. Then the MGS difference score was calculated between the baseline MGS score and the mean of MGS score at 30 min and 150 min after last anesthesia, respectively [21]. As the MGS difference scores did not significantly differ between the three observers, the MGS difference score of each mouse was averaged to be used for further statistics.

### Luxury behavior

Luxury behavior like burrowing and nest building behavior are only present when important needs of the mice are met and, thus, can serve as indicators of well-being [23].

#### Burrowing behavior

The test was modified according to Jirkof et al. [24]. A standard opaque plastic water bottle (250 ml, 150 mm length, 55 mm diameter, 45 mm diameter of bottle neck) was filled with 140 ± 2 g food pellets normally supplied as diet and placed parallel to the back wall of the photography cube. After 2 h, when the test ended, the food pellets [g] removed from the bottle by the mice were weighted.

#### Nest building behavior

The nests were scored by using a modified protocol developed by Deacon [25]. A nestlet (Ancare, Bellmore, NY, USA; UK agent: Lillico, Betchworth, UK) with an exact weight of 2.0 g was placed in the middle of the cage (Makrolon type III, 420 × 260 × 150 mm) bedded with 0.5 cm height of bedding material. In order to reduce distress caused by a new environment, used bedding material without feces from the home cage was scattered on top of the new bedding. No further environmental enrichment items were provided.

2 h after the light was turned on, the nests were assessed on a 5-point scale (1 = more than 90% of the nestlet intact; 2 = 50–90% intact; 3 = 50–90% shredded; 4 = more than 90% shredded but flat nest, less than 50% of its circumference is higher than mouse body height when curled up; 5 = more than 90% shredded and high nest, more than 50% of its circumference is higher than mouse body height when curled up) [25]. Any untorn nestlet pieces, defined as approximately 0.1 g, were weighted.

### Home cage activity

Home cage activity was evaluated by InfraMot (TSE systems, Bad Homburg Germany) during the dark period for 12 hours (6:00 p.m. to 6:00 a.m.). An infrared sensor was mounted on the top of the gridded cage top and recorded the number of impulses per minute. For analysis, impulse intervals of 10 min were used and the area under the time curve (AUC) [impulses/(10 min)] was calculated.

### Free exploratory paradigm

The free exploratory paradigm is a test to investigate trait anxiety-related behavior [26]. The gridded cage top was placed in the cage at an angle of 45° to the longer side of the home cage. Then, the latency to first exploration [s] (with all four paws on the lid) within 10 min was observed.

### Rotarod test

An accelerating rotarod was used to evaluate motor coordination and balance. The mice were placed on the rotating drum at a speed of 4 rounds per minute. The speed of the rotarod accelerated to 40 rounds per minute. The latency [s] to loose balance and fall off the rotating drum was measured. Some mice held on and rode around the rotarod a few times before they lost their balance. For those mice, the time until they finally fell off the rotarod was recorded. Mice performed 4 trials at 2 days after the last anesthesia, with a maximum of 300 s and 30-min intertrial rest intervals [5, 27]. Trial 1–3 served as training and trial 4 as the test trial.

### Food intake and body weight

Food intake of the standard food diet [g] was manually measured over a period of 24 hours. Since tiny pieces of food pellets may fall through the gridded cage top, the cage side beneath the food unit was carefully scanned.

The body weight [g] was regularly controlled over the experimental period. For analysis, the body weight of day 0, day 2, day 9, and day 14 after last anesthesia was used.

### Corticosterone

FCM with a 5α-3β,11β-diol structure in feces and hair corticosterone were measured since the sampling techniques are non-invasive. In our study, FCM indicate acute stress during the 24-hour postanesthetic period and hair corticosterone may reflect chronic stress. Since there are differences in baseline values, for each mouse the percentage change [%] relative to baseline was calculated.

#### FCM

The mice were single-housed for a period of 24 hours. In order to prevent distress due to a new cage, used bedding material without feces from their home cage was scattered on top of the new bedding. All dry fecal pellets were collected from the cages by using forceps. Wet pellets contaminated with urine were eliminated.

FCM were extracted from the 24-hour-bulk samples in accordance to Palme et al. [28]. Briefly, fecal samples were dried at a temperature of 60–70°C and then homogenized by a mortar. An aliquot of 0.05 g was shaken with 1 ml of 80% methanol for 30 min on a multi-vortex. After centrifugation (2500 x g, 15 min), 0.5 ml of supernatant was pipetted in an Eppendorf cup. Before and after extraction, the samples were stored at –80°C. The samples were analyzed for corticosterone metabolites using a 5α-pregnane-3b,11b,21-triol-20-one enzyme immunoassay as described and validated for mice by Touma et al. [29, 30].

#### Hair corticosterone

Hair was cut off with an electric shaver for small animals (Aesculap Isis GT 420, Suhl, Germany). Hair corticosterone [pg/mg] was analyzed by liquid chromatography-mass spectrometry in the laboratory of Prof. Kirschbaum, Department of Psychology, Technische Universität Dresden, Germany, as described previously [31].

### Statistical analysis

Statistical analysis was performed with IBM SPSS Version 23 (IBM Corporation, Armonk, NY, USA). Explorative data analysis and tests for normality were performed for each parameter. First, differences between female groups (control, single anesthesia, repeated anesthesia), secondly, differences between male groups (control, single anesthesia, repeated anesthesia), and, thirdly, sex differences (female versus male control, female versus male single anesthesia, female versus male repeated anesthesia) were analyzed using the respective test indicated in the result section.

In all tests, differences were considered significant at p < 0.05. In tables, data are presented as mean ± standard deviation, in graphs data are presented as mean ± standard error.

## Results

### Anesthesia

#### Phases of anesthesia

In female (U (13, 13) = 154.000, p < 0.001) and male (U (13, 13) = 146.000, p = 0.001) mice, repeated anesthesia significantly prolonged the duration of induction (Table 1). In addition, in female mice, repeated anesthesia prolonged the duration of non-surgical tolerance (U (13, 13) = 161.000, p < 0.001) and shortened the duration of surgical tolerance (U (13, 13) = 1.000, p < 0.001) versus a single anesthesia (Table 1).

**Table 1:** Phases of anesthesia.

Group	Induction [s]	Non-surgical tolerance [s]	Surgical tolerance [s]	Wake-up period [s]
**Single anesthesia ♀** (n = 13)	92.62 ± 20.13	107 ± 15.57^##^	2500.38 ± 30.17^##^	150.15 ± 98.99
**Repeated anesthesia ♀** (n = 13)	117.15 ± 8.07***	173.38 ± 50.21***	2431.00 ± 21.36***^,^ ^#^	105.38 ± 79.96
**Single anesthesia ♂** (n = 13)	100.85 ± 15.09	154.77 ± 37.25	2444.38 ± 47.06	126.31 ± 63.46
**Repeated anesthesia ♂** (n = 13)	117.38 ± 12.97**	151.46 ± 42.69	2452.69 ± 22.04	89.15 ± 72.04

Data are given as mean ± standard deviation. p values were calculated using Mann-Whitney-U-Test

** p < 0.01

*** p < 0.001 versus a single anesthesia

^#^ p < 0.05

^##^ p < 0.01 versus ♂.

Sex differences in mice with a single anesthesia were observed regarding the duration of non-surgical tolerance (U (13, 13) = 143.500, p = 0.002) and surgical tolerance (U (13, 13) = 27.000, p = 0.002) (Table 1). Non-surgical tolerance was significantly shorter and surgical tolerance significantly longer in female mice exposed to single anesthesia. In mice with repeated anesthesia, surgical tolerance was significantly shorter in female than in male mice (U (13, 13) = 132.000, p = 0.014).

#### Excitations, opisthotonus, and twitches

In female and male mice, repeated anesthesia increased excitations, versus a single anesthesia, during induction (female mice: Chi^2^ (1) = 7.8, p = 0.005; male mice: Chi^2^ (1) = 4.887, p = 0.027) but not during the wake-up period (female mice: Chi^2^ (1) = 0.377, p = 0.539; male mice: Chi^2^ (1) = 1.04, p = 0.308) (Table 2). Running excitement, a high muscle tonus of the tail, and movements of the tail were observed and defined as excitations. Opisthotonus (female mice: Chi^2^ (1) = 1.182, p = 0.277); male mice: Chi^2^ (1) = 2.167; p = 0.141) only occurred during the wake-up period and twitches (female mice: Chi^2^ (1) = 0.248, p = 0.619; male mice: Chi^2^ (1) = 0, p = 1) during the recovery period. The frequency of opisthotonus and twitches did not differ between the groups. No sex differences were found.

**Table 2:** Number of mice showing excitations, opisthotonus, and twitches.

Group	Induction	Wake-up perriod		Recovery period
	Excitation	Excitation	Opisthotonus	Twitches
**Single anesthesia ♀** (n = 13)	7	2	3	3
**Repeated anesthesia ♀** (n = 13)	13**	1	1	2
**Single anesthesia ♂** (n = 13)	7	0	2	1
**Repeated anesthesia ♂** (n = 13)	12*	1	0	1

Data are number of mice. p values were calculated using Chi-Square-Test

* p < 0.05

** p < 0.01 versus single anesthesia.

#### Vital parameters

The mean ± standard deviation were calculated from all four measurements of heart rate, oxygen saturation, and respiratory rate (Table 3). In female (t (20) = –2.687, p = 0.014) and male mice (t (21) = –3.595, p = 0.002), repeated anesthesia significantly increased heart rate, versus a single anesthesia. In both sexes, oxygen saturation (female mice: t (20) = 0.621, p = 0.542; male mice: t (21) = –0.053, p = 0.958) and respiratory rate (female mice: t (20) = –1.211, p = 0.240; male mice: t (21) = –2.000, p = 0.59) did not differ between single and repeated anesthesia. No sex differences in heart rate, oxygen saturation or respiratory rate were found.

**Table 3:** Vital parameters.

Group	Heart rate (bpm)	Oxygen saturation (%)	Respiratory rate (brpm)
**Single anesthesia ♀** (n = 9)	489.67 ± 31.65	98.51 ± 0.19	97.5 ± 15.12
**Repeated anesthesia ♀** (n = 13)	530.27 ± 36.83*	98.46 ± 0.21	105.08 ± 11.90
**Single anesthesia ♂** (n = 10)	483.08 ± 32.24	98.49 ± 0.30	91.38 ± 15.93
**Repeated anesthesia ♂** (n = 13)	549.06 ± 50.52**	98.5 ± 0.25	98.37 ± 12.34

Data are given as mean ± standard deviation. bpm, beats per min; brpm, breaths per min. p values were calculated using unpaired Student t-test

* p < 0.05

** p < 0.01 versus single anesthesia. Due to malfunction of the pulse oximeter, 4 female mice and 3 male mice of the single anesthesia group had to be excluded from statistics.

### Recovery period

Within the period of 20 min after the last anesthesia, repeated anesthesia significantly reduced the number of grooming episodes (U (13, 13) = 26.000, p = 0.030) and the duration of activity (t (19.907) = 2.116, p = 0.047) versus a single anesthesia in female mice (Table 4). By contrast, in male mice, repeated anesthesia increased the duration of food intake (t (21) = –5.435, p < 0.001) and decreased the duration of activity (t (21) = 3.726, p = 0.001) versus a single anesthesia (Table 4). Repeated anesthesia decreased the latency to the first food intake after the last anesthesia versus a single anesthesia in female mice (U (13, 13) = 31.500, p = 0.005) and male mice (U (13, 13) = 17.000, p < 0.001) (Table 4). Male and female mice did not significantly differ.

**Table 4:** Recovery period.

Group	Number of rearing^1^	Number of grooming episodes^1^	Duration of resting^1^ [s]	Duration of activity^2^ [s]	Latency to first food intake^1^ [s]	Duration of food intake^2^ [s]
**Single anesthesia ♀** (n = 9)	17 ± 12.11	33.67 ± 15.33	114.41 ± 185.61	581.41 ± 130.55	568.38 ± 265.18	296.15 ± 103.36
**Repeated anesthesia ♀** (n = 13)	23.15 ± 25.98	19.62 ± 8.70*	122.72 ± 190.63	441.55 ± 179.38*	351.54 ± 196.50**	411.16 ± 192.19
**Single anesthesia ♂** (n = 10)	27.4 ± 24.85	22.7 ± 11.99	132.26 ± 201.10	673.29 ± 151.75	557.85 ± 124.11	221.09 ± 110.62
**Repeated anesthesia ♂** (n = 13)	30.08 ± 30.04	16.38 ± 7.86	63.72 ± 107.71	469.36 ± 111.13**	342.92 ± 129.69***	500.43 ± 130.20***

Data are given as mean ± standard deviation. Recovery period was defined as the time from first forward movement until 20 min after the last anesthesia. Due to a malfunction of the camera, 4 female mice and 3 male mice of the single anesthesia group had to be excluded from the statistics. p values were calculated using Mann-Whitney-U-Test^1^ or unpaired Student t-test^2^.

* p < 0.05

** p < 0.01

**** p < 0.001 versus a single anesthesia.

### MGS

**Fig 2:**
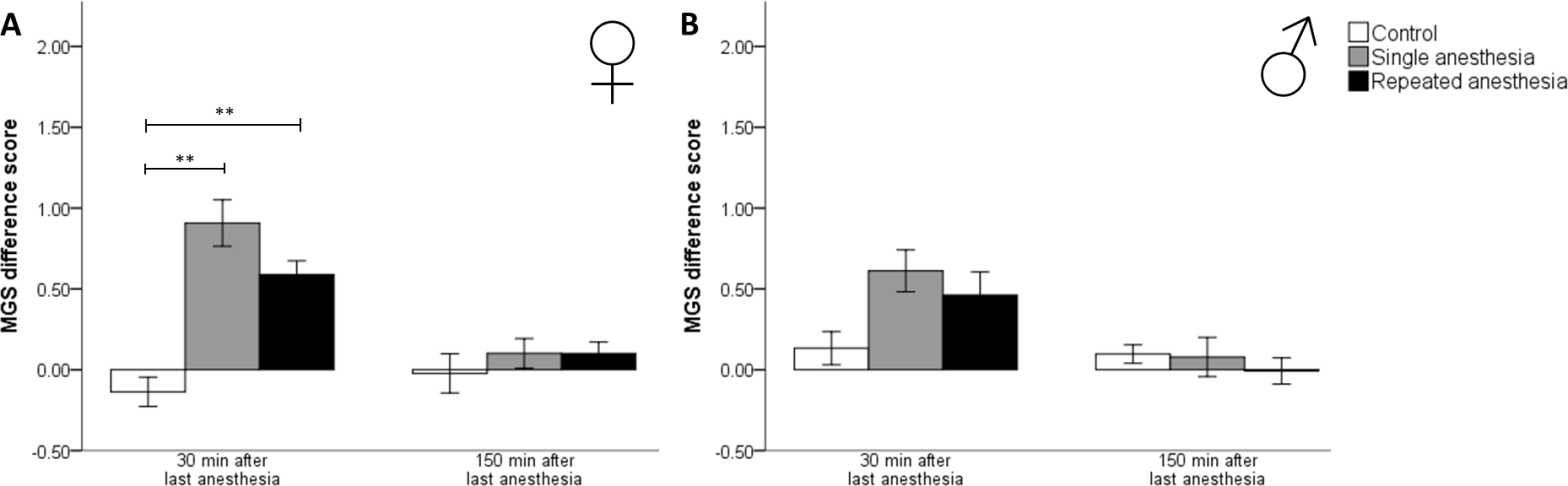
Mouse Grimace Scale difference scores at 30 min and 150 min after last anesthesia. MGS, Mouse Grimace Scale. Data are mean ± standard error. p values were calculated using Kruskal-Wallis-Test: ** p < 0.01. (A) Control ♀: n = 6, single anesthesia ♀: n = 9, repeated anesthesia ♀: n = 13; 4 mice of the single anesthesia group were excluded from statistics because of technical malfunction of the camera. (B) Control ♂: n = 6, single anesthesia ♂: n = 10, repeated anesthesia ♂: n = 13; 3 mice of the single anesthesia group and 1 control mouse were excluded from statistics because of technical malfunction of the camera.

30 min after last anesthesia, in female mice, both single (z = 3.467, p = 0.002) and repeated anesthesia (z = 3.019, p = 0.008) caused significantly higher MGS difference scores versus control (Fig 2A). At the following observation time point, 150 min after last anesthesia, the MGS difference scores no longer differed between female groups (Chi^2^ = 0.967, p = 0.617). No significant differences between male groups were observed either 30 min (Chi^2^ = 5.216, p = 0.074) or 150 min (Chi^2^ = 0.964, p = 0.618) after the last anesthesia (Fig 2B). No sex differences were found.

### Luxury behaviors

#### Burrowing behavior

**Fig 3:**
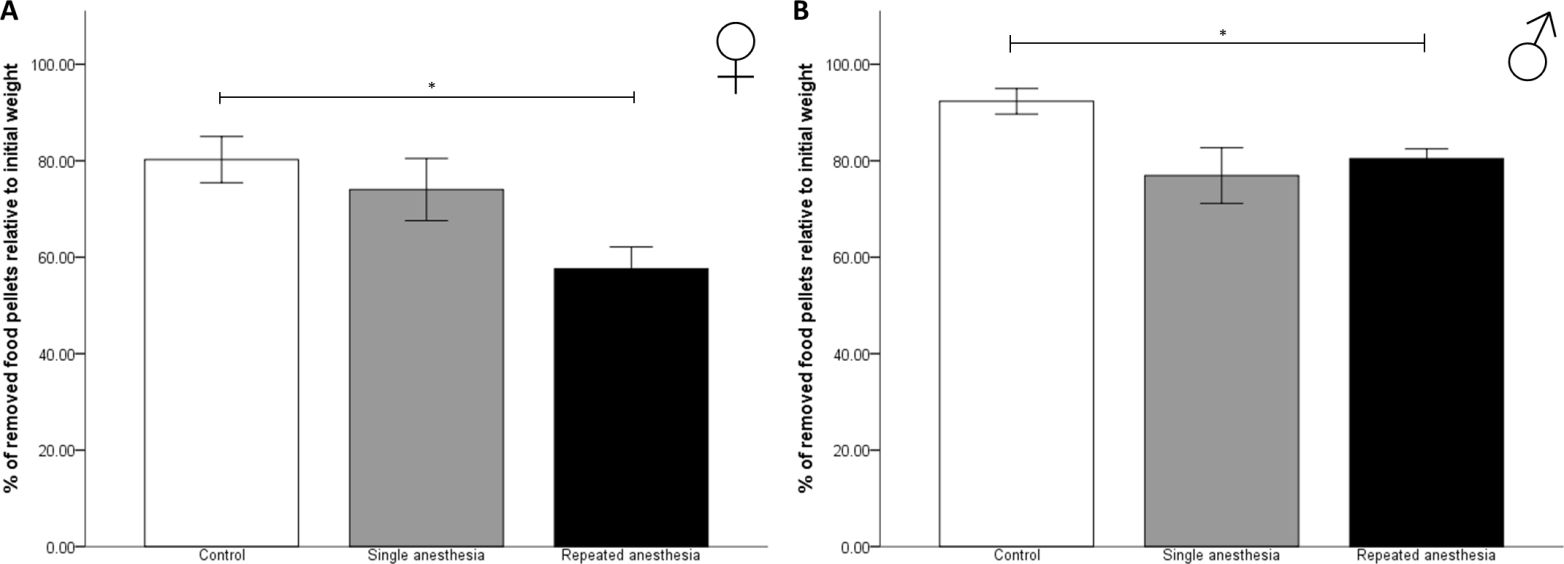
Burrowing behavior in the immediate postanesthetic period. Data are means ± standard error. p values were calculated using Kruskal-Wallis-Test: * p < 0.05. (A) Control ♀: n = 6, single anesthesia ♀: n = 9, repeated anesthesia ♀: n = 13; 4 mice of the single anesthesia group were excluded from statistics because of technical malfunctions of the scale. (B) Control ♂: n = 6, single anesthesia ♂: n = 10, repeated anesthesia ♂: n = 13; 3 mice of the single anesthesia group and 1 control mouse were excluded from statistics because of technical malfunction of the scale.

The weight of removed food pellets from the burrow relative to initial weight [%] was calculated. In female (z = –2.507, p = 0.036; Fig 3A) and male (z = –2.604, p = 0.028; Fig 3B) mice, repeated anesthesia significantly reduced the percentage of weight of removed food pellets versus control. Sex differences were found for repeated anesthesia, when female mice removed significantly less food pellets from the burrow than male mice (U (13, 13) = 151.000, p < 0.001).

#### Nest building behavior

Kruskal-Wallis-Analysis did not reveal any significant differences in the nest scores between female groups (values are mean ± standard deviation; control: 3.75 ± 1.47; single anesthesia: 4.58 ± 0.57; repeated anesthesia: 4.04 ± 1.09; Chi^2^ = 2.857, p = 0.240) and male groups (control: 4.36 ± 1.11; single anesthesia: 4.00 ± 0.91; repeated anesthesia: 4.27 ± 0.53; Chi^2^ = 2.076, p = 0.354), respectively. No sex differences were detected.

### Home cage activity

One day after the last anesthesia, the AUC [impulses^2^/(10 min)^2^] neither significantly differed between female groups (values are mean ± standard deviation; single anesthesia: 7.4 ± 4.1 M; repeated anesthesia: 7. 4 ± 3. 8 M; control: 8. 8 ± 2.7 M; Chi^2^ = 1.396, p = 0.498, Kruskal-Wallis-Test) nor between male groups (single anesthesia: 6.8 M ± 2.6 M; repeated anesthesia: 4.8 ± 1.9 M; control: 5.5 ± 1.8 M; Chi^2^ = 5.230, p = 0.073, Kruskal-Wallis-Test). The Mann-Whitney-U-Test revealed that control female mice moved significantly more than control male mice (U (6, 7) = 6.000, p = 0.035, Mann-Whitney-U-Test).

### Free exploratory paradigm

**Fig 4:**
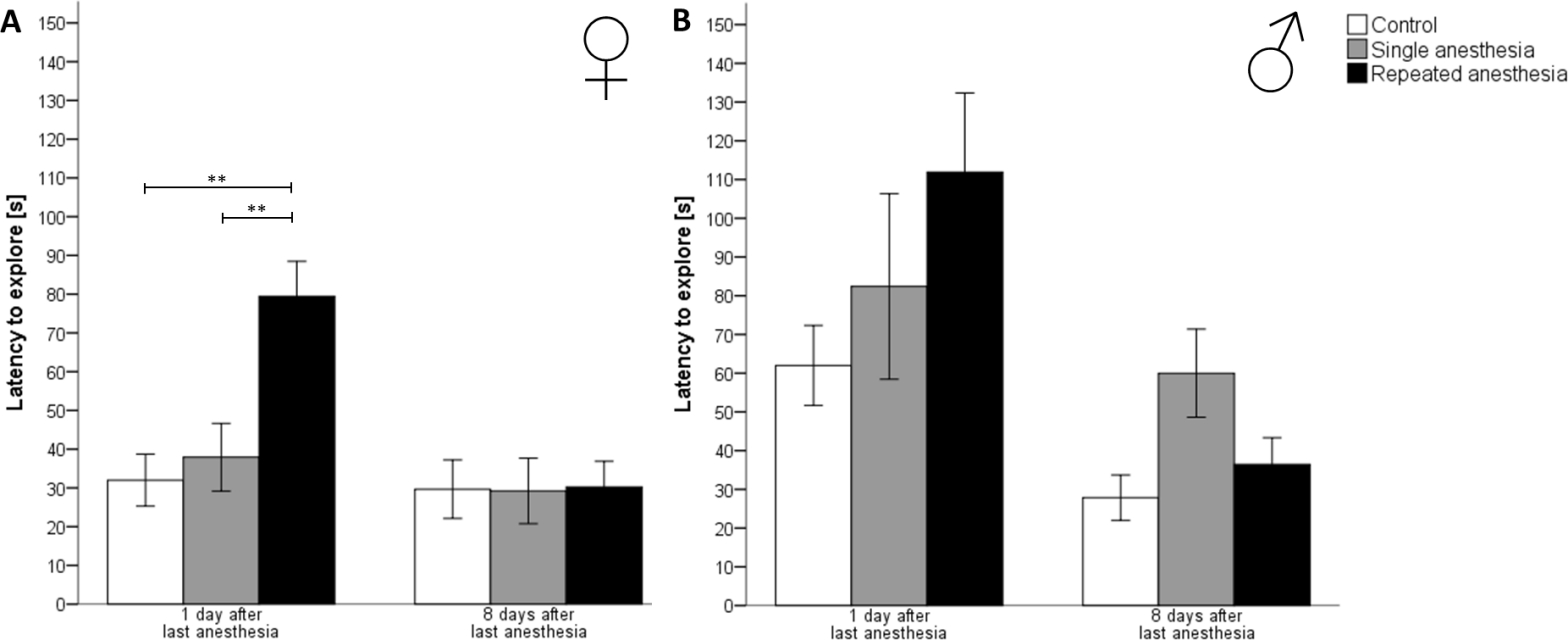
Latency to explore in the free exploratory paradigm for trait anxiety-related and exploratory behavior. Latency to explore [s]; data are means ± standard error; p values were calculated using the Kruskal-Wallis-Test: ** p < 0.01 versus repeated anesthesia. (A) Control ♀: n = 6, single anesthesia ♀: n = 13, repeated anesthesia ♀: n = 13. (B) Control ♂: n = 7, single anesthesia ♂: n = 13, repeated anesthesia ♂: n = 13.

Within the test period of 10 min, all mice explored the cage top. Kruskal-Wallis-Analysis revealed that, in female mice, repeated anesthesia significantly increased the latency to explore the cage top on the first day after anesthesia in comparison to control (z = 2.956, p = 0.009) and single anesthesia (z = –3.535, p = 0.001) (Fig 4A). 8 days after the last anesthesia, the latency to explore did no longer differ between female groups (Chi^2^ = 0.652, p = 0.722).

In male mice, the Kruskal-Wallis-Test revealed no significant differences in the latency to explore neither at 1 day (Chi^2^ = 2.525, p = 0.283) nor at 8 days (Chi^2^ = 3.404, p = 0.182) after last the anesthesia (Fig 4B).

Sex differences were found in the latency to explore. 1 day after the last anesthesia, in female control mice, the latency to explore was significantly shorter than in male control mice (U (6, 7) = 38.000, p = 0.014, Mann–Whitney-U-Test). 8 days after the last anesthesia, female mice given single anesthesia explored the gridded cage top significantly earlier than their male counterparts (U (13, 13) = 130.000, p = 0.019, Mann-Whitney-U-Test).

### Rotarod test

2 days after the last anesthesia, there were no differences in the rotarod performance in female groups (latency to fall given as mean ± standard deviation [s]; control: 285.17 ± 23.034; single anesthesia: 271.62 ± 60.98; repeated anesthesia: 297.17 ± 9.82; Chi^2^ = 3.227, p = 0.199, Kruskal-Wallis-Test) or male groups (control: 242.43 ± 86.32; single anesthesia: 250.92 ± 54.19; repeated anesthesia: 213.08 ± 101.74; Chi^2^ = 0.878, p = 0.645, Kruskal-Wallis-Test).

The Mann-Whitney-U-Test revealed sex differences in mice with repeated anesthesia: the latency to fall was significantly higher in male mice (U (13, 13) = 29.000, p = 0.007).

### Food intake and body weight

#### Food intake

One day after the last anesthesia, female mice given repeated anesthesia consumed significantly less food than female mice given single anesthesia (F (2, 29) = 3.457, p = 0.047) (Table 5). 8 days after the last anesthesia, female mice given repeated anesthesia consumed significantly more food than female controls (F (2, 29) = 9,965, p = 0.012) and female mice with a single anesthesia (F (2, 29) = 9,965, p = 0.001). In male mice, no significant differences in food intake between the groups were found at day 1 (F (2, 30) = 0.238, p = 0.79) and day 8 (F (2, 30) = 1.593, p = 0.22) after the last anesthesia.

**Table 5:** Food intake.

Group	Food Intake [g/g body weight]	
	1 day after last anesthesia	8 days after last anesthesia
**Control ♀** (n = 6)	0.22 ± 0.03	0.21 ± 0.02
**Single anesthesia ♀** (n = 13)	0.23 ± 0.05^##^	0.2 ± 0.03
**Repeated anesthesia ♀** (n = 13)	0.18 ± 0.06*	0.26 ± 0.04**^, +, ###^
**Control ♂** (n = 7)	0.18 ± 0.06	0.19 ± 0.02
**Single anesthesia ♂** (n = 13)	0.18 ± 0.03	0.21 ± 0.03
**Repeated anesthesia ♂** (n = 13)	0.19 ± 0.03	0.20 ± 0.03

Data are given as mean ± standard deviation. p values were calculated using ONEWAY ANOVA (post hoc Tukey-HSD)

* p < 0.05

** p < 0.01 versus single anesthesia

^+^ p < 0.05; versus control. p values were calculated using Student t-Test

^##^ p < 0.01

^###^ p < 0.001 versus ♂.

Sex differences existed in food intake at 1 day after the last anesthesia, when male mice given a single anesthesia consumed significantly less food (t (19.449) = 3.224, p = 0.004) than female mice given a single anesthesia; 8 days after the last anesthesia, male mice given repeated anesthesia ingested significantly less food (t (24) = 4.518, p < 0.001) than female mice given repeated anesthesia.

#### Body weight

**Fig 5:**
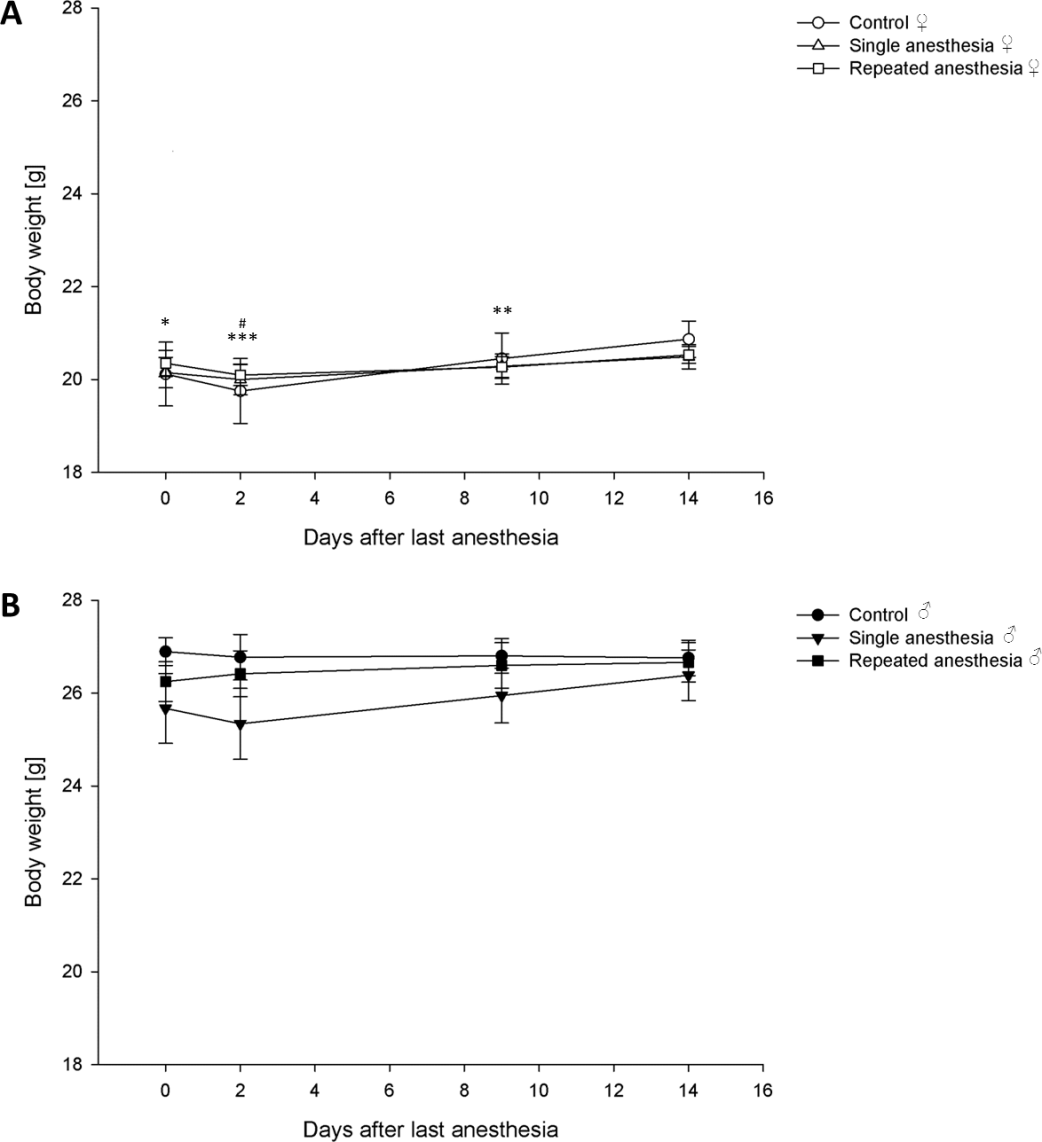
Course of body weight. Data are means ± standard error. (A) Control ♀: n = 6, single anesthesia ♀: n = 13, repeated anesthesia ♀: n = 13. (B) Control ♂: n = 7, single anesthesia ♂: n = 13, repeated anesthesia ♂: n = 13. p values were calculated using repeated measures ANOVA with group as between-subject factor: * p < 0.05, ** p < 0.01, *** p < 0.001 versus 14 days after the last anesthesia; ^#^ p < 0.05 versus 9 days after the last anesthesia.

The body weight was analyzed by performing repeated measures ANOVA with group as between-subject factor. Tests of within-subject comparison indicated that the group (females: F = 0.841, p = 0.538; males: F = 1.134, p = 0.35) had no effect on the course of body weight, but there was a significant time effect in female mice (females: F = 7.863, p < 0.001; males: F = 1.879, p = 0.153) (Fig 5). Pairwise comparisons revealed that the body weight of female mice was significantly higher at day 14 compared to day 0 (p = 0.013), 2 (p < 0.001), and 9 (p = 0.004) after the last anesthesia, and higher at day 9 compared to day 2 (p = 0.013) after the last anesthesia.

### Corticosterone

The percentage change of both FCM (female groups: Chi^2^ = 4.261, p = 0.119; male groups: Chi^2^ = 2.119, p = 0.347; Kruskal-Wallis-Test) and hair corticosterone (female groups: Chi^2^ = 2.228, p = 0.328; male groups: Chi^2^ = 0.689, p = 0.709; Kruskal-Wallis-Test) did not significantly differ in female and male groups, respectively (Table 6). No sex differences were observed.

**Table 6:** Fecal corticosterone metabolites (FCM) and hair corticosterone.

Group	FCM [%]	Hair corticosterone [%]
**Control ♀** (n = 6)	82.38 ± 31.36	73.43 ± 14.89
**Single anesthesia ♀** (n = 13)	115.54 ± 36.18	81.43 ± 29.75
**Repeated anesthesia ♀** (n = 13)	102.3 ± 50.14	91.34 ± 29.72
**Control ♂** (n = 7)	100.8 ± 20.80	96.25 ± 24.32
**Single anesthesia ♂** (n = 13)	117.97 ± 25.97	101.5 ± 50.50
**Repeated anesthesia ♂** (n = 13)	151.11 ± 89.31	89.5 ± 23.91

Data are given as mean ± standard deviation. The percentage change relative to baseline value was calculated. FCM, fecal corticosterone metabolites.

## Discussion

Within the scope of refinement, the goal of this study was to compare the effects of repeated with single isoflurane anesthesia on the well-being of adult C57BL/6JRj mice, in order to assess the severity of repeated isoflurane anesthesia. Well-being and distress were assessed by a behavioral test battery, the measurement of body weight, and non-invasive analysis of stress hormones in feces and fur. The behavioral test battery included a broad range of parameters, i.e. luxury behavior, the MGS, food intake, anxiety-related behavior, and motor activity. Moreover, the process of anesthesia was monitored, e.g. phases of anesthesia, motor effects, and vital parameters.

The main findings of the present study are that, depending on the sex, repeated isoflurane anesthesia caused short-term effects in a test of anxiety-related behavior, on the MGS, on burrowing behavior, and, with some caution, on food intake. Effects in the free exploratory paradigm were clear since mice with repeated anesthesia showed significantly higher anxiety-related behavior versus mice with single anesthesia and controls. The MGS indicated a difference between both repeated and single anesthesia versus control, though, no difference between single and repeated anesthesia. Effects on food intake were only seen between single and repeated anesthesia, but not in comparison to control. Burrowing behavior following repeated anesthesia was only decreased in comparison to control, but not when compared to single anesthesia. Interestingly, repeated anesthesia also causes an increase in excitations during induction compared to single anesthesia. Overall, our findings suggest that repeated isoflurane anesthesia had a slightly stronger impact on the stress level and the well-being of mice, especially of female mice, than single isoflurane anesthesia. Well-being seemed to be stabilized at day 8 after the last anesthesia, at the latest, indicated by the free exploratory paradigm.

In the free exploratory paradigm, all mice, independently from the group, explored the cage top. However, the latency to explore was higher in females, but not in males following repeated isoflurane anesthesia, suggesting a higher anxiety level. Our results are supported by observations of higher anxiety levels in female C57BL/6 mice in the elevated plus maze, a test for state anxiety [32]. A reason for the sex dependent effect may lie in the higher hypothalamic-pituitary-adrenal (HPA) axis activity and corticosterone release found in female rodents at baseline [33], in response to stress insults [34] and isoflurane exposure [35] compared to male rodents. Stress activates the HPA axis and increases glucocorticoid secretion. Glucocorticoids in turn activate retrograde endocannabinoid signaling to GABAergic (type A γ-aminobutyric acid) neurons and, hence, suppress synaptic inhibition [36, 37]. This mechanism increases excitability of neurons in the basolateral nucleus of the amygdala (BLA), which are implicated in anxiety-related behavior [37]. However, according to Long II et al., the GABAergic activity is increased in the BLA of rats after repeated isoflurane anesthesia, which is not compatible with elevated anxiety-related behavior [38].

Besides glucocorticoids, sexual hormones can also be responsible for the higher anxiety level in females. Both sexual hormones and stress hormones influence the 5-HT1A receptor, which plays an important role in modulating anxiety-related behavior [39] and coping with stressful situations [40]. Pieces of evidence suggest that isoflurane influences the functional state of the 5-HT1A receptor in male marmosets [41] and hippocampal 5-HT1A receptor binding in male rats at high concentrations [42]. The 5-HT1A receptor underlies sex differences with higher 5-HT1A receptor binding in female compared to male mice in particular brain regions (basolateral amygdala, claustrum, median raphe nucleus) [43]. In rats, estradiol suppresses 5-HT1A receptor signaling [44] and corticosterone decreases 5-HT1A mRNA in the dentate gyrus [45]. Accordingly, we hypothesized that isoflurane exposure might result in different changes of 5-HT1A receptor binding in females due to the higher estradiol and corticosterone levels, which may explain the higher anxiety levels found in female mice of our study. Moreover, in males, isoflurane inhibits 5-HT uptake in a noncompetitive manner [42], decreases extracellular 5-HT in the mouse hippocampus [46] and decreases 5-HT release in the rat frontal cortex [47]. Since Rex et al. demonstrated a relationship between anxiety-related behavior and 5-HT release in the ventral hippocampus [48], it would be of particular interest to compare the effects of isoflurane on the brain 5-HT levels in females and males.

We applied the MGS at 30 min and 150 min after the last anesthesia, which allows us to analyze the sole impact of anesthesia and not the pharmacological effects of isoflurane. This is based on the knowledge that only 0.2% of isoflurane is metabolized. Isoflurane is eliminated from the brain of rabbits according to the pharmacokinetic two-compartment model with half-lives of 26 min and 174 min [49]. Since the metabolism is faster in mice than rabbits, we assumed that at 30 min and 150 min after anesthesia, most of the inhaled isoflurane was already eliminated from the body so that any pharmacological effects of isoflurane on the behavior of mice and the MGS could be excluded. Both single and repeated isoflurane anesthesia caused a short-term mild increase of the MGS difference scores in the immediate postanesthetic period indicating that repeated anesthesia did not augment the effect of a single application. However, this effect was statistically significant in female mice only, which indicated, that especially the well-being of female C57BL/6JRj mice seemed to be affected by the exposure to isoflurane. Our findings are in line with Miller and Leach revealing that, 30 min after a single isoflurane anesthesia of 10 min, the MGS is increased in CBA mice [50]. There is only little data available investigating the impact of repeated anesthesia on the Grimace Scale. A recent study by Miller et al. [51] shows that in male rats, the Grimace Scale scores recorded after anesthesia are increased when the rats are repeatedly anesthetized with isoflurane (12 min over a 4-day period). Our study is the first to show a comparable effect in mice. We could demonstrate that 150 min after a single or the last anesthesia, at the latest, the MGS difference scores return to control level.

The increase of MGS scores may be due to the noxious stimulus of isoflurane. Its smell is described as pungent [52] and the inhalation of 2.3% isoflurane evokes coughing, burning, irritation, and other discomfort in humans [53]. Similar responses can be expected in mice. It is possible that nociceptive ion channels, transient receptor potential ankirin 1 (TRPA1) and vanilloid 1 (TRPV1), which are activated by isoflurane, enhance the noxious stimulus of isoflurane and potentiate neurogenic inflammation and pain [54]. The sexual dimorphism found in the MGS with significant higher scores in females, but not in males after both single and repeated isoflurane anesthesia versus control may be explained by sex differences in pain nociception as demonstrated by Sorge et al [55]. Overall, the results of the MGS indicate that both single and repeated isoflurane anesthesia impaired well-being, especially, of female C57BL/6JRj mice in the immediate postanesthetic period. We suggest that the impact on well-being can be rated as mild since the MGS difference scores after anesthesia remained < 1 and no significant differences between a single and repeated isoflurane anesthesia were found.

The display of luxury behavior like burrowing and nest building behavior serves as an indicator of well-being in mice [23]. In our study, repeated isoflurane anesthesia reduced burrowing behavior versus controls in mice of both sexes, which was more pronounced in females. However, because of the lack of a significant difference between single and repeated anesthesia, this effect was only weak. Accordingly, repeated anesthesia slightly negatively affected well-being in mice of both sexes during the immediate postanesthetic period. This observation is in line with the work of Jirkof et al. with sevoflurane [24]. However, in contrast to Jirkof et al. [56], who described a reduction of nest building behavior following a short mono-anesthesia with sevoflurane, nest building behavior was not affected in our study. One reason for the discrepancy could be that Jirkof et al. applied a different nest complexity scoring as they carried out the scoring already 9 hours after anesthesia, i.e. at the end of the light period. We decided to score the nests not before 14 hours after the last anesthesia, i.e. in the morning of the following day after the dark period, according to the protocol by Deacon [25]. Therefore, we believe that mice had more time to build complex nests, since high nest scores are achieved especially at the end of the dark phase [56].

Food intake and body weight are useful as non-invasive parameters to assess postoperative distress, well-being as well as appetite in mice. Although mice do not vomit, they can suffer from postoperative nausea, which was proven to be triggered by sevoflurane in mice [57]. Whereas neither single nor repeated anesthesia had a significant effect on the course of body weight in mice of both sexes, female mice showed a marginally decreased food intake 1 day after the last repeated anesthesia, but 8 days afterwards a slightly increased feeding behavior, maybe as some kind of compensation mechanism. This is underlined by the fact that female mice did not significantly lose, but put on weight over time. In accordance to our results, Jacobsen et al. found no change in body weight but, across days, a significant change in food intake of mice that underwent isoflurane anesthesia with or without vasectomy [58]. Hence, analysis of food intake and body weight indicated no impairment of the well-being of male mice, but short-term mild impairment of the well-being of female mice. Differences in food intake between mice with a single anesthesia and mice with repeated anesthesia are result of slightly different effects compared to control.

Both single and repeated isoflurane anesthesia did not increase hair corticosterone levels, independent from sex. Hair corticosterone was described as a retrospective biomarker to reliably reflect long-term HPA axis activity in humans [59], but also in rodents [60]. Whereas our anesthesia protocol did not elevate hair corticosterone levels, other studies have proven the association of a stress insult and an increase in hair corticosterone concentration in mice, e.g. social defeat [61] and social instability [62]. Taking the effects of these stress insults on hair corticosterone into account, our anesthesia protocol did not seem to cause chronic stress.

Similar to hair corticosterone, neither single nor repeated isoflurane anesthesia elevated FCM in both female and male mice. The same method of FCM analysis has been previously utilized in mice and a correlation between an increase in FCM levels and various stressors was found (e.g. train-induced vibrations [63], oral gavage [64], and blood sampling [65]). The peak of the fecal corticosterone metabolite level is reached 8–10 h after a stressor depending on the intestinal transit time from duodenum to rectum, which is influenced by the activity of the mice [29]. Since neither single nor repeated isoflurane anesthesia decreased the home cage activity during the dark period following the last anesthesia, we assumed that the peak was not delayed. In order to prevent any influence of the expressed circadian rhythm on the excretion of FCM [30], samples were collected over a period of 24 h, i.e. all dry fecal pellets were collected. Overall, especially our FCM results, but also the lack of an increase in hair corticosterone levels indicate that our anesthesia protocols did not cause an expressed HPA axis response in the 24-hour postanesthetic period and later on. However, for future studies, it would be of interest to analyze the momentary stress by blood sampling.

The analysis of the anesthesia phases revealed that repeated anesthesia prolonged the duration of induction in mice of both sexes and increased excitations. Li et al. [66] reported that isoflurane caused airway irritation and neurogenic constriction by the activation TRPA1 channels, which impaired respiratory function and prolonged the duration of induction. The activation of TRPA1 channels may have been enhanced by repeated isoflurane anesthesia with prolongation of induction. However, future studies are necessary to clarify this hypothesis. In mice of both sexes, repeated isoflurane anesthesia increased the heart rate. This may be explained by the observation that repeated exposure to isoflurane is more aversive than initial exposure, which might have led to an elevated arousal in mice [7] and, subsequently, an increase in heart rate. The increase in heart rate and duration of induction may indicate that mice developed a reduced sensitivity to isoflurane due to re-exposure, which was already reported for other volatile anesthetics [67]. Since prolonged exposure to both isoflurane or diazepam reduces sensitivity to isoflurane due to the cross-sensitivity [68], both agents are suggested to act via the GABA_A_ receptor, the major inhibitory neurotransmitter receptors [69]. Therefore, the GABA_A_ receptor may play a central role in the development of a reduced sensitivity to isoflurane. Further investigation is needed regarding the subunit involved, e.g. via knock-in/knock-out mice [70].

The behavioral test battery was feasible for the assessment of well-being in the postanesthetic period, although the analysis of stress hormones in feces and fur seemed to be unsuitable for measuring postanesthetic stress. With the objective of refinement, we assume that our behavioral test battery will also be useful to investigate the influence of other procedures performed in animal experimentation.

## Conclusion

Repeated isoflurane anesthesia caused short-term mild distress and reduced well-being mainly in the immediate postanesthetic period. Regarding our anesthesia protocol, repeated isoflurane anesthesia in C57BL/6JRj mice can be classified as mild. However, sex differences have to be taken into account, as the well-being of female mice seems to be more affected by isoflurane anesthesia.

Within the mild severity category, depending on the sex, repeated isoflurane anesthesia ranks higher than single isoflurane anesthesia. The findings of the present study should be known, when the overall severity level of an animal experiment is estimated by adding the severity levels of all procedures being performed in the course of an experiment, which includes repeated isoflurane anesthesia.
